# Radiomorphology of the Habib Sealer-Induced Resection Plane during Long-Time Followup: A Longitudinal Single Center Experience after 64 Radiofrequency-Assisted Liver Resections

**DOI:** 10.1155/2010/403097

**Published:** 2010-08-30

**Authors:** Robert Kleinert, Roger Wahba, Christoph Bangard, Klaus Prenzel, Arnulf H. Hölscher, Dirk Stippel

**Affiliations:** ^1^Department of General, Visceral and Cancer Surgery, University of Cologne, 50923 Cologne, Germany; ^2^Center of Integrated Oncology, University of Cologne, 50923 Cologne, Germany; ^3^Department of Radiology, University of Cologne, 50923 Cologne, Germany

## Abstract

*Background*. Radiofrequency (RF-) assisted liver resection devices like the Habib sealer induce a necrotic resection plane from which a small margin of necrotic liver tissue remains in situ. The aim of the present paper was to report our long-time experience with the new resection method and the morphological characteristics of the remaining necrotic resection plane. *Methods*. 64 RF-assisted liver resections were performed using the Habib sealer. Followup was assessed at defined time points. *Results*. The postoperative mortality was 3,6% and morbidity was 18%. The followup revealed that the necrotic zone was detectable in all analyzed CT and MRI images as a hypodense structure without any contrast enhancement at all time points, irrespectively of the time interval between resection and examination. *Conclusion*. Liver resection utilizing radiofrequency-induced resection plane coagulation is a safe alternative to the established resection techniques. The residual zone of coagulation necrosis remains basically unchanged during a followup of three years. This has to be kept in mind when evaluating the follow up imaging of these patients.

## 1. Introduction

Surgical resection is currently the best therapeutic option in many liver malignancies, especially in colorectal liver metastases, the most frequent indication for liver resection in western countries. Intraoperative blood loss is one of the predictive factors for a postoperative outcome [1], particularly in multiple atypical resections for metastases. New techniques like the Habib sealer (Habib 4X sealer) were developed with the objective to minimize the risk of parenchyma bleeding and therefore reduce the perioperative risk [2]. Although, the RFA-assisted liver resection using the Habib sealer is well established in the daily liver surgery, there are only a few long-time follow-up reports about this procedure [3] and consequently only little information about the morphological and biological effects of the remaining necrotic margin. Up to now, it is not clear what happens to this necrotic tissue; one possible scenario is that parts of the zone might be decomposed and resorbed in the course of healing, as known from zone of ablation after radiofrequency ablation of hepatic tumors [4, 5]. Another scenario is that the remaining margin is not decomposed and remains as a necrotic barrier in situ. To evaluate local tumor recurrence or possible complications surgeons and radiologists who are involved in the usage of this technique have to know the immediate and long-term morphological characteristics of the necrotic resection plane in up to date imaging techniques after liver resection.

The aim of the present paper was to present our experience with radiofrequency-assisted liver resection using the Habib 4X sealer in particular with regard to the radio-morphological characteristics of the Habib sealer-induced necrotic resection plane.

## 2. Materials and Methods

### 2.1. Patients

Between June 2005 and August 2008-64 RF assisted liver resections were performed in 56 patients by using the Habib 4X sealer at the Department of General, Visceral and Cancer Surgery of the University of Cologne. All patients underwent standard preoperative assessment of their disease [6], including spiral computed tomography (CT) scanning or magnetic resonance imaging (MRI), and showed no evidence of unresectable extrahepatic disease. The most frequent indications for liver resection were colorectal liver metastasis (CRM) followed by hepatocellular carcinoma and metastasis from various other diseases (breast cancer, sarcoma, thyroid cancer). 4 patients underwent surgery for nonmalignant diseases: one with liver rupture, two patients with haemangioma, and one patient with echinococcosishydatid cyst, making liver resection necessary. 56 atypical liver resections and 8 hemihepatectomies (right *n* = 5, left *n* = 3) were performed. The mean age was 62 years (range 32 to 89 years). The indications and resection types are summarized in Table 1.

### 2.2. Procedure

**Figure 1:**
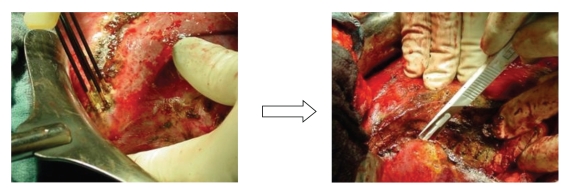
Probes were positioned in the liver parenchyma. A necrosis of healthy parenchyma measuring 1 cm in width was induced. The liver parenchyma was then dissected with a scalpel near the proximal margin of the 1-cm wide necrotic zone.

Under general anaesthesia, a modified right subcostal incision was made. The peritoneal cavity was examined to exclude any extrahepatic disease. The liver was mobilized according to the size and site of the lesion. An intraoperative ultrasound was performed before liver resection for detailed planning of the resection plane and to reveal potentially undetected lesions. The Habib 4X sealer (RITA Medical Systems, USA) consists of an array of four bipolar radiofrequency probes that are connected to a generator (Rita generator model 1500X, RITA Medical Systems, Mountain View, CA). It uses a modification of the principle of radiofrequency ablation (RFA). The bipolar electrodesare was inserted into the liver tissue along the intended line of parenchymal dissection. A high-frequency current causes tissue heating, which leads to a coagulative necrosis and thereby shrinkage of proteins. This procedure creates a 1- to 3-cm wide coagulative resection plane between and around the electrodes. After coagulating the parenchyma of the intended resection plane, it is dissected with a scalpel in the middle of the coagulated resection plane as described by the Habib group [2] (Figure 1). The applied output varied from 75 to 250 Watt depending on the localization in the liver. The higher the electric output, the shorter the time to shutoff, which is automatically done by the generator, when the impedance increases by a certain margin after the initial decrease in impedance. The shorter the time of energy deposition, the narrower the coagulation zone will be, due to less timerequiredfor conductive heat transport. The liver parenchyma was then dissected with a scalpel near the tumor facing margin of the necrosis plane. Resection margins were examined for bleeding, and bleeding vessels or visible bile ducts were sutured.

### 2.3. Analysis

Intraoperative data were assessed prospectively by recording predefined parameters in a standardized premade study protocol: parameters included patient data (age, gender, and diagnosis) and statistical data about the operation (operation time, resection time, and blood loss). Furthermore, and the resected specimen and size of the resection plane were measured and weighted before the specimen was put on formaldehyde. 

Short-time followup was assessed postoperatively (POP) at defined time points (1, 3, 5, and 7 days) and included clinical examination and blood sampling. Long term followup was done retrospectively: clinical status was assessed by interviewing the patient and the patients' family doctor. CT (with intravenous applied contrast agent) and MRI scans were analyzed at different time points (1 to 36 month). CT and MRI scans were analyzed with Mevislab image processing and visualization software (Mevis Medical Solution AG, Bremen, Germany), which allows the determination of dimension and volumes (liver, necrotic zone). Radiomorphological diagnostic and measurements were supervised by an experienced radiologist. All resected specimens went to pathology examination. Dimensions, weight, size of the resection plane, and tumor safety margins were determined.

### 2.4. Computer Aided Measurements of the Necrotic Margin

**Figure 2:**
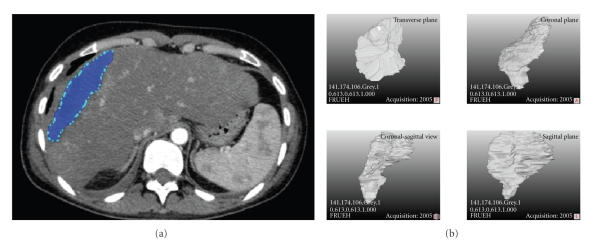
The necrotic zone (marked blue) was isolated using an edge detection-based segmentation algorithm and rendered to a three-dimensional object (left image). The four images on the left side represent the object from different views.

The CT scans were imported into Mevislab. A segmentation algorithm based on different grey values allowed an edge detection of the necrotic zone and thus the identification of the boundaries. Based on the voxel values (*x*, *y*, and *z*), the necrotic zone was isolated from the original CT scan and rendered to a three dimensional object (Figure 2). In the next step a morphometric analysis of the object was performed. 

The main axes of the three-dimensional (3D) object were determined, and the thickness for each Z position was determined in an angle of 90 degrees. Thus, Mevislab allowed the determination of the maximum, minimum, and mean thickness of the object.

### 2.5. Statistical Analysis

Values are expressed as mean ± SD, unless otherwise stated. Data were analyzed using the SPSS software package version 15.0. (SPSS Software GmbH, Munich, Germany).

## 3. Results

In 56 patients, 64 RF-assisted liver resections were performed using the Habib 4X sealer. Followup period was between 4 and 36 months. All patients and the family doctors were interviewed by telephone. 36 patients survived the observation period. Among the oncological patients (52 patients), 32 patients survived the observation period.

Mean surgery time was 146 minutes (±51 minutes) whereas mean time for parenchymal dissection was 21 minutes (±12 minutes). The thickness of the resected lesions varied from0.7 cmto 14 cm (mean 4.0 ± 2.0 cm). The weight of the resected specimen varied from 8.2 g to 834 g (mean 227 g), and the size of the resection plane varied from 15 cm² to 184 cm² (mean 68 cm²). 19 patients showed macroscopic signs of liver cirrhosis. In 11 patients, additional RFA was performed to treat multifocal liver metastases. A pringle maneuver was performed to stop the blood supply in 10 patients (8 to 23 minutes). Additional surgery was performed in 20 patients: thirteen cholecystectomies, four colon resections, one nephrectomy, one adrenalectomy and one partial diaphragm resection. Mean blood loss during whole procedure was in the range of 200 mL to 1280 mL (mean 630 mL with a standard deviation of 375 mL. There were significant differences in the blood loss between cirrhotic livers (mean 857 ± 370 mL) and noncirrhotic livers (mean 400 ± 250 mL) (see Table 2). 

### 3.1. Morbidity and Mortality

Mean postoperative hospital stay was 14 days. All patients were observed on the intensive care unit (ICU) and 90% of the patients were dismissed from the ICU within the first 24 hours. Two patients (3.6%) died postoperatively. One patient with the diagnosis of hepatocellular carcinoma in advanced liver cirrhosis developed fulminate liver failure on the second postoperative day after right trisegmentectomy and died of liver insufficiency. The other death was caused by a small bowel perforation of unknown origin. The patient died due to peritonitis on the 37th postoperative day. Complications were registered in 10 patients (17.9%), as shown in Table 3. There were 3 patients with severe complications: one patient suffered from postoperative hemorrhage of the resection plane, which made a relaparotomy necessary; a second patient developed a heparin-induced thrombocytopenia and consequently a thrombus in the thoracic aorta, and the third patient suffered from postoperative lung embolism. These three patients were treated successfully and were dismissed from the hospital in good clinical condition after a prolonged postoperative course. Moderate complications were observed in 5 (9%) patients: bilioma developed in three patients (5.6%). These patients required percutaneous drainage (14, 28 and 34 days resp.) and antibiotic drug therapy for one week. Two patients showed pleural effusion, which made a temporary percutaneous drainage necessary. Transfusion was limited to 6 patients (10.8%). Among the transfused patients were the two patients who died and the three patients with severe complications mentioned above. In addition, the patient with a traumatic liver rupture needed transfusion.

The safety margins measured by the pathologist were compared to the postoperative outcome and the underlying disease. Mean safety margin was 0.5 cm (0.1 cm to 3.5 cm). During the followup no patient had evidence for a local recurrence, even those with a safety margin of 0.1 cm in the specimen. There was no correlation between recurrence of the underlying tumor disease and the size of the resection margin. The average safety margin in the disease-free surviving group was not significantly higher than in the patients who died because of their underlying tumor disease (Table 4).

### 3.2. Morphology of the Resection Plane

**Figure 3:**
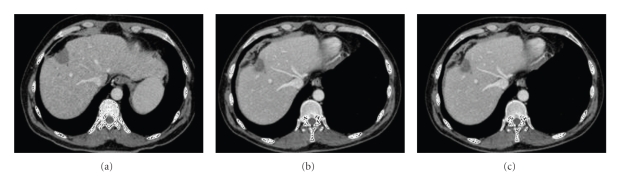
CT scans 15 days (a), 6 months (b), and 20 months (c) after liver resection for HCC in cirrhotic liver.

**Figure 4:**
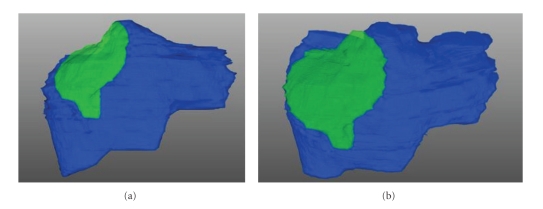
3D reconstruction of the liver 15 days (a) and 20 months (b) after liver resection for HCC. The necrotic margin is yellow colored and is not detached during regeneration.

In totally, 37 CT and 11 MRI scans were available and were analyzed (Table 5). In all CT and MRI images, a hypodense structure without any contrast enhancement was detectable at the site of resection. Figure 3 shows an example of the morphology of the necrotic residuum of the same patient at different time points (three, six, and twenty months, resp.). The necrotic margin is visual at all time points, irrespectively of the time interval between resection and examination. The three-dimensional computer reconstructions in Figure 4 visualize that; although the liver is regenerating, the necrotic margin seems to be constant over the whole process and that the margin was not decomposed. This assumption could be verified by the computer-assisted measurements of the necrotic margin (see also Table 5): 3 to 6 months after resection, the mean thickness of the necrotic margin was 4.3 cm while 9 to 12 and 18 to 24 month after resection the size remained almost unchanged (3.6 cm and 3.9 cm). There was a high variation of the thickness of the necrotic zone between the patients, as there is known correlation between applied energy and size of the necrotic margin [7].

## 4. Discussion

Radiofrequency-assisted liver resection in the treatment of liver malignancies is a new concept. There are already several studies which verify that this method is safe [2, 3]. The resection plane shows the characteristic of an absolutely dry and homogeneous necrotic tissue with obliterated bile ducts and vessels. As bleeding on the resection plane is a potential postoperative complication after liver resection [8], the question arises whether the necrotic margin is acting as a tissue barrier. Subsequently, the risk of intraoperative bleeding should be reduced. Our study underlines this theory as mean blood loss in noncirrhotic and cirrhotic livers was comparable to previous studies dealing with the Habib technique [9] and conventional liver resection [10]. Furthermore, small bile ducts are obliterated during the coagulation process, and subsequently early postoperative bilioma should be reduced. In our series, postoperative bilioma was seen in 5.6% which is lower than in conventional liver resection where rates from around 7% [11, 12] up to 13% [13] are described. However, the duration of the observed bile fistulas was rather prolonged in comparison to other modalities of hepatic parenchymal dissection, as the patients required percutaneous drainage for up to 34 days.

A comparative analysis of morbidity and mortality in patients undergoing conventional versus RFA-assisted hepatic resection is difficultto performbecause a standard manner to report postoperative complications is lacking. Moreover, our patients—like in most of the published studies—involved cirrhotic as well as noncirrhotic patients with different hepatic disorders such as fibrosis, cholestasis, or hepatitis. Thus, reported postoperative complications of patients undergoing conventional liver resection vary between 9% [11] and 67% [14] depending on the extent of resection and comorbidity. In our patients, overall morbidity was 18%, and mortality was 4%. Although strict patient selection may reduce the postoperative mortality to nearly 0%, most of the studies report a mortality especially caused by postoperative hepatic failure up to 5% [13–16].

Up to now there are no reports about the biological and morphological effects of the remaining necrotic margin. On the biological side, some authors raise the question whether the remaining necrotic margin favors a superinfection of the necrotic margin [17]. Although none of our 52 patients showed clinical signs for a superinfection of the necrotic zone in terms of subhepatic abscess, a higher number of cases may be necessary to prove this theory.

The creation of the necrotic margin raises another question: is direct ablation using radiofrequency ablation (RFA) a well-established method to destroy malignant tumors up to 3 cm [18, 19]? As RFA causes a necrotic defect of the liver which is verifiable by histology, the hypothesis is emerging whether the resection plane is simultaneously acting as an oncological safety plane, as potential micrometastasis are destroyed with the liver tissue. This could enlarge the resection margin in patients submitted to hepatic resection in addition to the resection margin measured in the specimen. Our study underlines this assumption, as in our series of 64 tumor resections, there was no local recurrence, although mean safety margin was 0.5 cm. (0.1 cm to 3.5 cm). Ultimate proof to this hypothesis could of course only be gained by histopathological samples of the necrotic zone in all patients, which is ethically impossible.

The morphological analysis revealed that the necrotic zone was not decomposed during liver regeneration and is detectable in CT scans even 36 months after resection. This is an essential fact, as the presence of a hypodense structure might mislead unaware radiologists and surgeons to a false diagnosis of a local recurrence or new tumor.

## 5. Conclusion

Liver resection using radiofrequency induced resection plane coagulation is a safe alternative to the established resection techniques. The residual zone of coagulation necrosis remains basically unchanged during a followup of two years. This has to be kept in mind when evaluating the followup imaging of these patients.

## Figures and Tables

**Table 1:** Indications and surgical procedures.

Indication	Atypical resection	Right hemihepatectomy	Left hemihepatectomy		Total resections	
Colorectal liver metastasis	27	2	2		31	(= 48%)
Other liver metastasis	9	—	—		9	(= 14%)
HCC	16	1	1		18	(= 28%)
CCC	1	1	—		2	(= 3%)
Hemangiomas	1	1	—		2	(= 3%)
Traumatic liver rupture	1	—	—		1	(= 2%)
Echinococcus cyst	1	—	—		1	(= 2%)

Total	56	5	3	0	64	100

**Table 2:** Intraoperative results.

Resection	Resections	OP time	Resection Time	Blood loss	Pringle	Additional surgery	Additional RFA	Pringle time	Mortality	Morbidity
	*n*	minutes	minutes	mL	*n*	*n*	*n*	minutes	*n*	*n*
(<1 segment)	47	112	18	410	7	17	7	9.6	2	8
(i) in noncirrhotic livers	33	95	16	253	5	15	3	8	2	4
(ii) in cirrhotic livers	14	121	21	680	2	2	4	11	0	4

(>2 Segments)	9	136	30	700	3	3	2	14	0	2
(i) in noncirrhotic livers	6	121	28	262	2	3	2	9	0	0
(ii) in cirrhotic livers	3	155	31	1283	1	0	0	23	0	2

Hemihepatectomy	8	162	33	676	0	0	2	0	0	2
(i) in non cirrhotic livers	7	149	25	685	0	2	2	0	0	2
(ii) in cirrhotic liver	1	240	75	610	0	1	0	0	0	0

**Table 3:** Morbidity and mortality.

Morbidity	*n*	%
Severe complications		
Fulminante liver failure	1	1.79
Small bowel perforation	1	1.79
Vascular occlusion	2	3.58
Hemorrhage	1	1.79

Moderate complications		
Bilioma	3	5.37
Pleura effusion	2	3.58

Total	10	17.9

Mortality	*n*	%

<30 Days		
Fulminante liver failure	1	1.79
Sepsis due to peritonitis	1	1.79

Total	2	3.58

>1 month to 2 years		
Disseminated tumor disease	17	30.43

**Table 4:** Safety margin = distance between tumor and resection plane.

Indication	Patients	Safety margin average (cm)	Safety margin range	

Colorectal liver metastasis	31	0.5	0.1 to 3.5 cm	
Other liver metastasis	9	0.6	0.1 to 0.5 cm	
HCC & CCC	20	0.4	0.1 to 2.0 cm	

Mortality	Patients	Safety margin average (cm)	Safety margin range	

Alive	24	0.6 ± 0.5	0.1 to 2.0 cm	
Dead due to tumor disease	32	0.5 ± 1.0	0.1 to 3.5 cm	

Total	56	0.5	0.1 to 3.5 cm	

**Table 5:** Mean thickness of the necrotic area in CT and MRI.

Examination interval	1 to 4 weeks	3 to 6 months	9 to 12 months	18 to 24 months	30 to 36 months	*n*
Number of examinations	8	9	13	13	5	48
Analyzed CT scans	8	5	9	11	4	37
Analyzed MRI scans	0	4	4	2	1	11

Mean thickness of necrotic area	3.75	4.3	3.6	3.89	3.85	
Standard deviation	1.27	1.45	1.38	1.44	2.4	

## References

[1] Bismuth H, Castaing D, Garden OJ (1989). Major hepatic resection under total vascular exclusion. *Annals of Surgery*.

[2] Weber J-C, Navarra G, Jiao LR, Nicholls JP, Jensen SL, Habib NA (2002). New technique for liver resection using heat coagulative necrosis. *Annals of Surgery*.

[3] Milićević M, Bulajić P, Žuvela M, Dervenis C, Basarić D, Galun D (2007). A radiofrequency-assisted minimal blood loss liver parenchyma dissection technique. *Digestive Surgery*.

[4] Curley SA (2001). Radiofrequency ablation of malignant liver tumors. *Oncologist*.

[5] Gillams AR, Lees WR (2005). Radiofrequency ablation of colorectal liver metastases. *Abdominal Imaging*.

[6] Grundmann RT, Hermanek P, Merkel S (2008). Diagnosis and treatment of colorectal liver metastases—workflow. *Zentralblatt fur Chirurgie*.

[7] Stippel DL (2007). Percutaneous, laparoscopic and open surgical radiofrequency ablation of malignant liver lesions. *Zentralblatt fur Chirurgie*.

[8] Gurusamy KS, Samraj K, Davidson BR (2007). Routine abdominal drainage for uncomplicated liver resection. *Cochrane Database of Systematic Reviews*.

[9] Ayav A, Jiao LR, Habib NA (2007). Bloodless liver resection using radiofrequency energy. *Digestive Surgery*.

[10] Habib N, Zografos G, Dalla Serra G, Greco L, Bean A (1994). Liver resection with total vascular exclusion for malignant tumours. *British Journal of Surgery*.

[11] Erdogan D, Busch ORC, van Delden OM, Rauws EAJ, Gouma DJ, van Gulik TM (2008). Incidence and management of bile leakage after partial liver resection. *Digestive Surgery*.

[12] Tanaka S, Hirohashi K, Tanaka H (2002). Incidence and management of bile leakage after hepatic resection for malignant hepatic tumors. *Journal of the American College of Surgeons*.

[13] Coelho JCU, Claus CMP, Machuca TN, Sobottka WH, Gonçalves CG (2004). Liver resection: 10-year experience from a single institution. *Arquivos de Gastroenterologia*.

[14] Midorikawa Y, Kubota K, Takayama T (1999). A comparative study of postoperative complications after hepatectomy in patients with and without chronic liver disease. *Surgery*.

[15] Poon RTP, Fan ST, Lo CM (2002). Extended hepatic resection for hepatocellular carcinoma in patients with cirrhosis: is it justified?. *Annals of Surgery*.

[16] Zhou X-D, Tang Z-Y, Yang B-H (2001). Experience of 1000 patients who underwent hepatectomy for small hepatocellular carcinoma. *Cancer*.

[17] Stavrou GA, Tzias Z, Von Falck C, Habib N, Oldhafer K-J (2007). Hepatic resection using heat coagulative necrosis. First report of successful trisegmentectomy after hypertrophy induction. *Langenbeck’s Archives of Surgery*.

[18] Mulier S, Ni Y, Jamart J, Ruers T, Marchal G, Michel L (2005). Local recurrence after hepatic radiofrequency coagulation: multivariate meta-analysis and review of contributing factors. *Annals of Surgery*.

[19] Navarra G, Ayav A, Weber J-C (2005). Short- and long-term results of intraoperative radiofrequency ablation of liver metastases. *International Journal of Colorectal Disease*.

